# Suppression of LPS-Induced Inflammation by *Phragmites communis* Young Leaf Extract via Multi-Target Inhibition of IκB, AP-1, and STAT1/3 Pathways in RAW 264.7 Cells

**DOI:** 10.3390/plants14142178

**Published:** 2025-07-14

**Authors:** Kyung-Yun Kang, Kyung-Wuk Park

**Affiliations:** R&D Team, Suncheon Research Center for Bio Health Care, Suncheon-si 57962, Republic of Korea; nms-kang@nate.com

**Keywords:** *Phragmites communis* leaves, therapeutic potential, anti-inflammatory, herbal medicine, flavonoids

## Abstract

Young leaves of reed (*Phragmites communis*) have been reported to exhibit antioxidant effects; however, their anti-inflammatory properties have not yet been investigated. In this study, we evaluated the effects of young reed leaf extract (PCE) on LPS-induced inflammation in RAW 264.7 cells and elucidated the underlying molecular mechanisms. Our results demonstrate that PCE significantly inhibited the production of nitric oxide (NO) by approximately 45% at 100 μg/mL (*p* < 0.01) and pro-inflammatory cytokines such as IL-6, TNF-α, and GM-CSF by 40–60% (*p* < 0.01) in LPS-stimulated RAW 264.7 macrophages, without cytotoxicity up to 100 μg/mL. PCE also downregulated the expression of inducible nitric oxide synthase (iNOS) and cyclooxygenase-2 (COX-2) and upregulated heme oxygenase-1 (HO-1) expression by approximately 2-fold at 100 μg/mL (*p* < 0.05). Mechanistically, these effects were associated with the inhibition of IκBα phosphorylation/degradation, IKKα/β phosphorylation, and AP-1 activation via the suppression of JNK and ERK signaling pathways, as well as the inhibition of STAT1/3 phosphorylation. Collectively, our findings suggest that PCE exerts anti-inflammatory effects by modulating the IκB, AP-1, and STAT1/3 signaling pathways, thereby suppressing inflammatory mediator production and enhancing antioxidant defense mechanisms in LPS-treated macrophages.

## 1. Introduction

Inflammation is a vital component of the immune system’s defense mechanism, acting as a protective response to harmful stimuli such as pathogens, toxins, and tissue injury [1]. While acute inflammation plays an essential role in initiating tissue repair and eliminating invading microbes, prolonged or chronic inflammation can become detrimental [2,3]. Chronic inflammation is increasingly recognized as a key contributor to the development and progression of numerous diseases, including cancer, cardiovascular disease, diabetes, neurodegenerative conditions, and autoimmune disorders [4,5,6]. This prolonged inflammatory state often involves sustained production of pro-inflammatory mediators, resulting in significant tissue damage and the disruption of homeostatic functions [7].

Among the critical mediators involved in inflammation are nitric oxide (NO), prostaglandin E2 (PGE_2_), and various cytokines, such as tumor necrosis factor-alpha (TNF-α), interleukin-1 beta (IL-1β), and interleukin-6 (IL-6) [8,9,10]. Produced primarily by activated macrophages, these molecules play essential roles in amplifying inflammatory responses [11,12]. However, excessive or dysregulated production is closely linked to chronic inflammation and tissue degeneration, making the modulation of their signaling pathways an important therapeutic target [1,7,13].

Lipopolysaccharide (LPS), a major component of Gram-negative bacterial membranes, is widely used to induce inflammatory responses in macrophages such as RAW 264.7 cells, activating key inflammatory pathways including nuclear factor-kappa B (NF-κB), mitogen-activated protein kinases (MAPKs), and the Janus kinase/signal transducer and activator of transcription (JAK/STAT) signaling cascades [14,15]. These pathways regulate the expression of inflammatory genes such as inducible nitric oxide synthase (iNOS), cyclooxygenase-2 (COX-2), and multiple pro-inflammatory cytokines [16]. In particular, the NF-κB pathway plays a central role: under basal conditions, NF-κB remains sequestered in the cytoplasm by inhibitor IκB. Upon LPS stimulation, IκB is phosphorylated by IκB kinase (IKK), leading to IκB degradation and the nuclear translocation of NF-κB, which in turn promotes the transcription of inflammatory genes [17]. Similarly, the MAPK pathway (including c-Jun N-terminal kinase [JNK] and extracellular signal-regulated kinase [ERK]) regulates transcription factors like activator protein-1 (AP-1), while the JAK/STAT pathway—particularly STAT1 and STAT3—modulates inflammatory gene expression [16].

*Phragmites communis* (common reed), an aquatic plant widely distributed across many regions, has traditionally been used in herbal medicine for its purported antioxidant, anti-inflammatory, and antimicrobial properties [18,19]. Previous studies have reported notable biological activities of *Phragmites communis*. For example, treatment with *Phragmites communis* leaf extract was shown to alleviate melanogenesis by reducing oxidative stress in B16F10 melanoma cells [20]. Additionally, the extract of *Phragmitis rhizoma* (the root of *Phragmites communis*) significantly improved docetaxel-induced myelotoxicity in hematopoietic progenitor cells [21]. In particular, young leaves of *Phragmites communis* have recently attracted attention due to their higher levels of bioactive compounds compared to mature plants [22]. Ethnobotanical knowledge supporting the medicinal use of reed plants provides a rationale for exploring their mechanisms of action in modern scientific contexts, particularly concerning inflammation [23]. Despite accumulating evidence regarding their antioxidant activities, the anti-inflammatory effects of young reed leaves—especially in macrophage-mediated inflammation models—remain underexplored [23].

Previous research has demonstrated that the inhibition of these pathways effectively reduces the production of inflammatory mediators [24]. Based on the antioxidant and traditional medicinal properties of *Phragmites communis*, it is hypothesized that extracts from its young leaves may exert anti-inflammatory effects by modulating these critical signaling networks [25]. Thus, the present study aims to investigate the anti-inflammatory properties of young reed leaf extract (PCE) in an LPS-induced RAW 264.7 macrophage model. Specifically, we evaluate its ability to suppress the production of inflammatory mediators (NO, cytokines, and PGE_2_) and to modulate key inflammatory signaling pathways, including NF-κB/IκB, AP-1, and STAT1/3 [24,26]. Through these investigations, we aim to provide foundational evidence supporting the potential of *Phragmites communis* shoots as a sustainable, plant-derived source for pharmaceutical, nutraceutical, and cosmetic applications.

## 2. Results and Discussion

### 2.1. Quantification of Flavonoids and Phenolics

The concentrations of total phenolic compounds and flavonoids in PCE were determined based on standard calibration curves constructed with gallic acid and quercetin, respectively. Quantitative results for each compound are summarized in Table 1.

Quantitative analysis showed that the PCE contained 4.14 ± 0.60 μg gallic acid equivalents (GAE)/mg of total phenolic compounds and 10.18 ± 1.29 μg quercetin equivalents (QE)/mg of flavonoids.

The results demonstrate that *Phragmites communis* leaf extract (PCE) contains notable amounts of total phenolics and flavonoids, indicating its potential as a natural source of antioxidant compounds. The levels of phenolic and flavonoid compounds are comparable to those found in other grass-derived and aquatic plants, such as Miscanthus sinensis and various aquatic grasses [27,28].

Phenolic and flavonoid compounds are widely recognized for their free radical-scavenging activity, primarily through electron donation, and have been linked to antioxidant and anti-inflammatory effects [29,30]. The results highlight the potential utility of PCE in the development of functional foods or nutraceuticals.

### 2.2. Effects of PCE on Cell Viability and LPS-Induced Nitric Oxide Production in Macrophages

**Figure 1:**
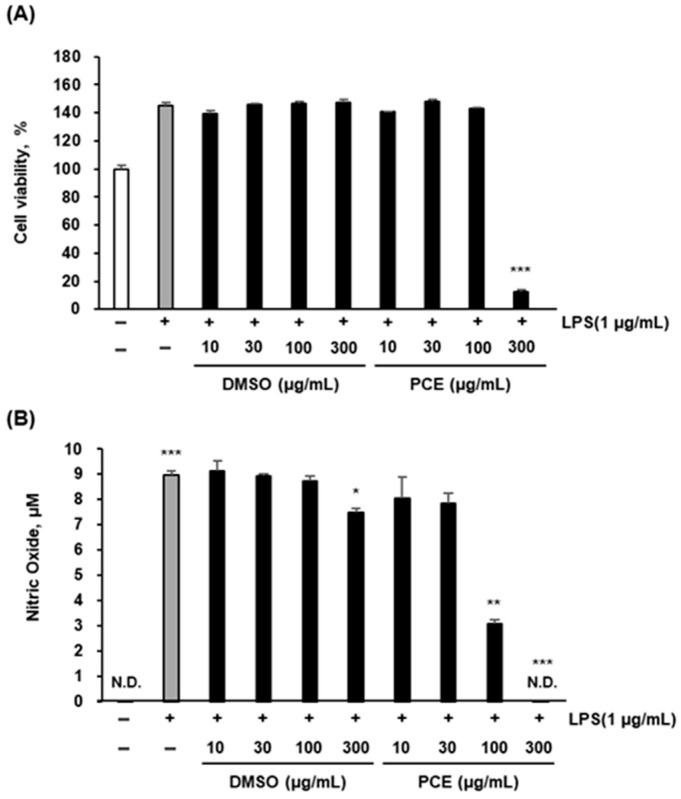
The effect of PCE on cell viability (**A**) and NO production (**B**) in LPS-stimulated RAW 264.7 macrophages. RAW 264.7 cells were treated with 1 μg/mL LPS for 1 h before treatment with PCE (10, 30, 100, and 300 μg/mL). The culture supernatant was then applied to the analysis of nitric oxide. RAW 264.7 cells were treated with PCE (10, 30, 100, and 300 μg/mL) for 24 h. The culture supernatant was removed, and the Cell Counting Kit-8 (CCK-8) solution was added. Results are expressed as mean ± SD from three independent experiments. Statistical significance was evaluated using one-way ANOVA followed by post hoc analysis (* *p* < 0.05, ** *p* < 0.01, *** *p* < 0.001); N.D.: not detected.

To assess the cytotoxic potential of PCE, RAW 264.7 macrophages were exposed to increasing concentrations of PCE (10, 30, 100, and 300 μg/mL) for 24 h, followed by cell viability analysis using the CCK-8 (Cell Counting Kit-8) assay. As presented in Figure 1A, PCE did not significantly alter cell viability at concentrations of 10, 30, or 100 μg/mL, indicating an absence of cytotoxicity at these doses. In contrast, a marked reduction in viability was observed at 300 μg/mL (*** *p* < 0.001), suggesting that this higher concentration induces cytotoxic effects. Furthermore, when PCE was dissolved in dimethyl sulfoxide (DMSO) and applied at concentrations below 100 μg/mL, no cytotoxic effects were observed, confirming DMSO as an appropriate solvent for subsequent in vitro experiments.

To further investigate the anti-inflammatory properties of PCE, nitric oxide (NO) production was measured in LPS-stimulated RAW 264.7 macrophages using the Griess reaction. As shown in Figure 1B, PCE treatment led to a concentration-dependent decrease in NO production. Statistically significant reductions were observed at 100 μg/mL (** *p* < 0.01) and 300 μg/mL (*** *p* < 0.001) relative to the LPS-only control group. However, the substantial inhibition of NO at 300 μg/mL is likely influenced by the cytotoxicity observed at this concentration rather than reflecting a purely pharmacological anti-inflammatory effect.

Taken together, these results suggest that PCE is non-cytotoxic at concentrations up to 100 μg/mL and effectively attenuates LPS-induced NO production in macrophages, indicative of its anti-inflammatory potential. The suppression of NO—a critical inflammatory mediator synthesized by inducible nitric oxide synthase (iNOS) upon LPS stimulation—implies that PCE may downregulate key pathways involved in inflammatory responses. These findings are consistent with previous reports demonstrating the anti-inflammatory efficacy of plant-derived extracts in macrophage-based in vitro models without significant cytotoxicity [8,31,32].

Given the cytotoxic response at 300 μg/mL, careful interpretation is required when evaluating anti-inflammatory outcomes at this concentration. Consequently, subsequent mechanistic studies in this work were conducted using concentrations ≤ 100 μg/mL to exclude potential confounding effects due to cell toxicity. These results provide foundational evidence supporting the therapeutic applicability of PCE in the treatment and management of inflammation-related disorders.

### 2.3. PCE Attenuates LPS-Induced Inflammatory Cytokine Production and Enhances HO-1 Expression in Macrophages

**Figure 2:**
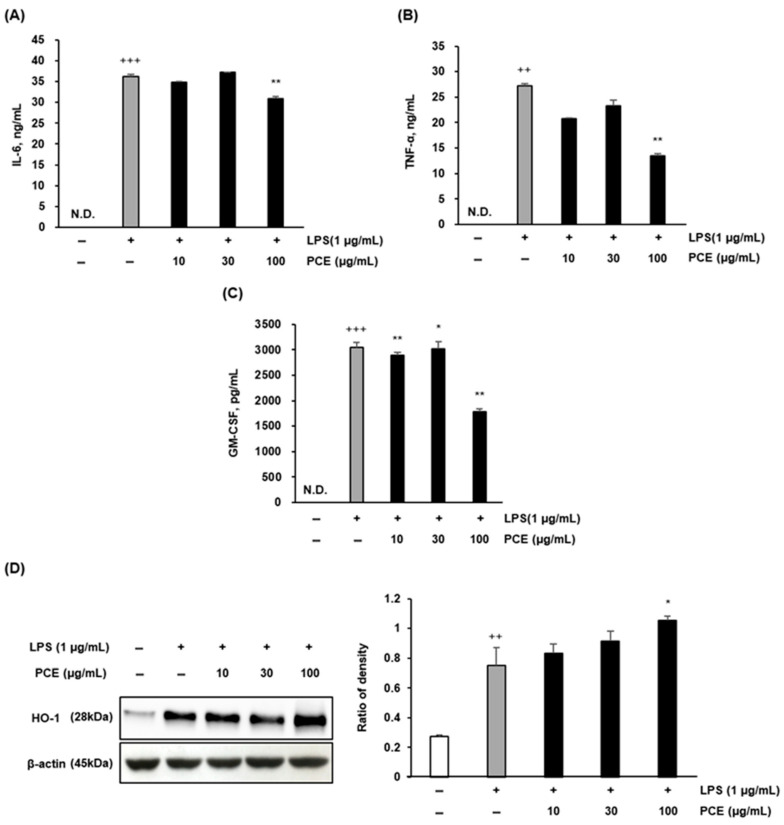
The effect of PCE on cytokine secretion in LPS-stimulated RAW 264.7 macrophages. RAW 264.7 cells were cultured with LPS 1 h before treatment with various types of PCE for 24 h, and the amount of pro-inflammatory cytokines (**A**–**C**) and HO-1 expressions (**D**) is shown. Data are expressed as mean ± SD from three independent experiments. Statistical analysis was performed using one-way ANOVA followed by post hoc testing. Significance is indicated as ++ *p* < 0.01 and +++ *p* < 0.001 versus the control group, and * *p* < 0.05, ** *p* < 0.01, versus the LPS-treated group; N.D.: not detected.

We evaluated the secretion of pro-inflammatory cytokines and the expression of antioxidant enzymes in LPS-stimulated RAW 264.7 macrophages treated with PCE. As shown in Figure 2A, PCE treatment effectively decreased cytokine secretion in a dose-dependent manner. Specifically, IL-6 and TNF-α levels were significantly suppressed at 100 μg/mL (** *p* < 0.01), while GM-CSF was significantly reduced at both 30 and 100 μg/mL (** *p* < 0.01) (Figure 2B,C). These findings suggest that PCE attenuates LPS-induced inflammation by downregulating the key cytokines involved in macrophage activation.

Cytokines like IL-6 and TNF-α play central roles in initiating and propagating inflammation, and their overexpression is associated with chronic diseases such as rheumatoid arthritis, atherosclerosis, and metabolic syndrome [33,34]. The suppression of these cytokines by PCE aligns with previous studies demonstrating that natural bioactive compounds can modulate immune responses and mitigate cytokine-mediated tissue injury.

Additionally, we examined PCE’s impact on cellular antioxidant defenses by assessing the expression of heme oxygenase-1 (HO-1), an enzyme known for its cytoprotective and anti-inflammatory properties. Western blot analysis (Figure 2B) revealed a significant dose-dependent increase in HO-1 expression, with the most pronounced upregulation observed at 100 μg/mL (* *p* < 0.05). This suggests that PCE may exert anti-inflammatory effects through the activation of endogenous antioxidant pathways, contributing to redox homeostasis.

Together, these results support the dual mechanism of PCE’s anti-inflammatory action: (1) the suppression of pro-inflammatory cytokine secretion (IL-6, TNF-α, GM-CSF) and (2) the upregulation of HO-1 expression. This dual action highlights PCE’s potential to target both inflammatory mediators and oxidative stress responses, two key features of chronic inflammation.

Prior studies have shown that the activation of HO-1 by natural products can inhibit NF-κB and MAPK signaling pathways, further suggesting that HO-1 may be a critical target of PCE’s action [35]. These findings provide strong support for the hypothesis that PCE confers anti-inflammatory and antioxidant benefits through a synergistic modulation of cytokine signaling and stress-response pathways.

### 2.4. PCE Inhibits LPS-Induced PGE_2_ Production and Downregulates iNOS and COX-2 Expression in Macrophages

**Figure 3:**
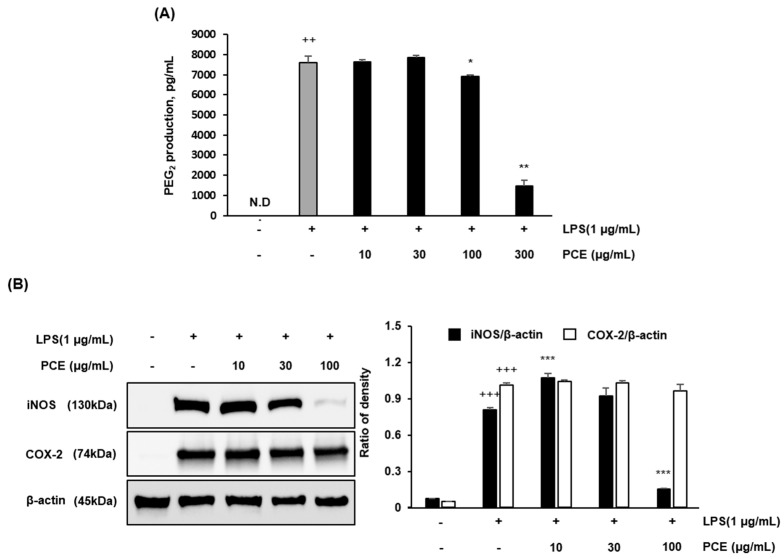
The effect of ethanol extractions of PCE on the expression of PGE_2_ (**A**) and iNOS, COX-2 (**B**) in LPS-stimulated RAW 264.7 macrophages. RAW 264.7 cells were cultured with LPS for 1 h before treatment with PCE (10, 30, and 100 μg/mL) for 24 h. After 24 h, lysates of the cells were subjected to Western blot analysis with iNOS, COX-2, and β-actin antibodies. Data are presented as mean ± SD from three independent experiments. Statistical significance was assessed using one-way ANOVA followed by post hoc testing (* *p* < 0.05, ** *p* < 0.01, *** *p* < 0.001 vs. control; ++ *p* < 0.01, +++ *p* < 0.001 vs. LPS); N.D.: not detected.

To elucidate the anti-inflammatory mechanisms of *Phragmites communis* extract (PCE), we investigated its effects on prostaglandin E_2_ (PGE_2_) production and the expression of two key inducible enzymes—inducible nitric oxide synthase (iNOS) and cyclooxygenase-2 (COX-2)—in LPS-stimulated RAW 264.7 macrophages. PCE resulted in a concentration-dependent suppression of PGE_2_ production (Figure 3A). However, PGE_2_ levels at 300 μg/mL are likely to be influenced by the cytotoxicity observed at this concentration rather than reflecting a purely pharmacological anti-inflammatory effect.

Western blot analysis results showed that PCE almost inhibited iNOS expression at the highest concentration (100 μg/mL) (*** *p* < 0.001), whereas COX-2 showed a tendency to decrease at the concentration of 100 μg/mL, but it was not significantly reduced (Figure 3B).

These findings demonstrate that PCE exerts anti-inflammatory effects by targeting multiple inflammatory mediators. The concurrent suppression of PGE_2_ and iNOS highlights the ability of PCE to modulate several key pathways involved in macrophage-mediated inflammation.

The downregulation of iNOS expression provides further support for the anti-inflammatory efficacy of PCE. iNOS is upregulated in response to pro-inflammatory stimuli and generates large amounts of nitric oxide (NO), which contributes to oxidative damage and tissue injury in chronic inflammation [36,37]. The observed suppression of iNOS by PCE indicates a possible mechanism by which PCE mitigates nitrosative stress and restores redox homeostasis in activated macrophages. Interestingly, these mechanistic effects parallel those of other well-characterized polyphenolic compounds, such as curcumin, resveratrol, and quercetin, which exert anti-inflammatory activity through the downregulation of COX-2 and iNOS via the inhibition of NF-κB and MAPK signaling pathways [38,39]. Based on these observations, the present findings suggest that PCE may exert its anti-inflammatory effects through multi-targeted modulation of inducible inflammatory enzyme pathways, thereby supporting its potential application as a natural therapeutic agent for the prevention or treatment of chronic inflammation-associated diseases.

### 2.5. PCE Suppresses Inflammation via IκB Pathway Inhibition in Macrophages

**Figure 4:**
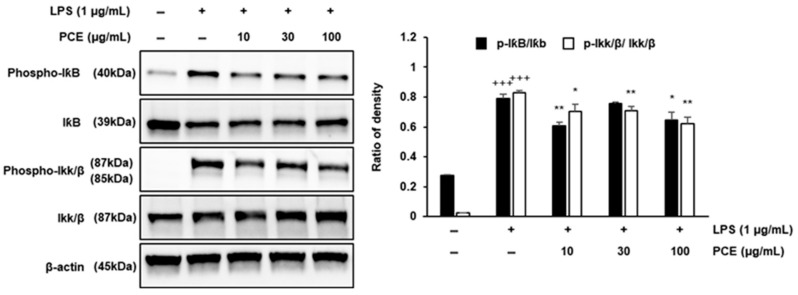
The effect of PCE on the expression of the IκB signaling pathway was determined by Western blot analysis in LPS-stimulated RAW 264.7 macrophages. RAW 264.7 cells were cultured with LPS for 1 h before treatment with PCE (10, 30, and 100 μg/mL) for 3 h. Data are presented as mean ± SD from three independent experiments. Statistical significance was evaluated using one-way ANOVA followed by post hoc analysis (* *p* < 0.05, ** *p* < 0.01 vs. control; +++ *p* < 0.001 vs. LPS).

To investigate the inhibitory effects of PCE on the IκB signaling pathway, we analyzed the expression of related signaling molecules in LPS-stimulated RAW 264.7 macrophages using Western blotting (Figure 4).

Treatment with PCE led to a dose-dependent inhibition of pathway activation. Specifically, PCE at concentrations of 10, 30, and 100 μg/mL significantly reduced the levels of phosphorylated IκB (phospho-IκB) and phosphorylated IKKα/β (phospho-IKKα/β) (*p* < 0.01), indicating the suppression of IKK activity and the prevention of IκB degradation. These findings suggest that PCE effectively attenuates the LPS-induced activation of the IKK/IκB signaling axis in RAW 264.7 cells. The inhibition of this pathway is likely a key mechanism underlying the anti-inflammatory effects of PCE, as the IκB/NF-κB pathway plays a central role in regulating the transcription of various pro-inflammatory genes, including TNF-α and IL-6 [40,41].

Our results are consistent with previous studies demonstrating that plant-derived compounds can modulate IκB signaling and exert anti-inflammatory effects in macrophages [42]. Taken together, these findings suggest that PCE may serve as a promising natural therapeutic agent for controlling inflammation through inhibition of the canonical IκB signaling pathway.

### 2.6. PCE Inhibits Inflammation via STAT Pathway Suppression in Macrophages

The STAT signaling pathway plays a pivotal role in regulating immune responses and inflammation. The phosphorylation of STAT proteins is promoted by pro-inflammatory cytokines elevated upon LPS stimulation, and these activated STATs subsequently contribute to the progression and amplification of inflammatory responses [43,44].

**Figure 5:**
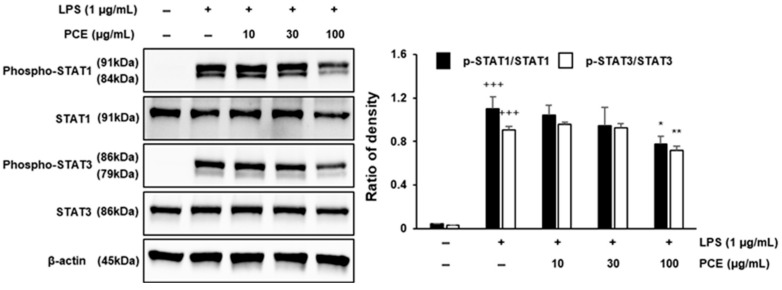
The effect of PCE on the expression of signal transducers and activators of transcription (STAT) in LPS-stimulated RAW 264.7 macrophages. RAW 264.7 cells were cultured with LPS for 1 h before treatment with PCE (10, 30, and 100 μg/mL) for 3 h. After 24 h, lysates of the cells were subjected to Western blot analysis with specific antibodies. Data are presented as mean ± SD from three independent experiments. Statistical analysis was performed using one-way ANOVA followed by post hoc testing (* *p* < 0.05, ** *p* < 0.01 vs. control; +++ *p* < 0.001 vs. LPS).

PCE treatment led to a dose-dependent inhibition of STAT1 and STAT3 phosphorylation (Figure 5). In particular, treatment with 100 μg/mL PCE significantly decreased the phosphorylation levels of both STAT1 and STAT3 in cells exposed to LPS (*p* < 0.05 or *p* < 0.01), without markedly affecting the total protein levels of STAT1 and STAT3. These findings indicate that PCE selectively inhibits STAT activation rather than altering STAT protein expression.

The observed inhibition of STAT phosphorylation suggests that PCE may play a crucial role in attenuating inflammatory cytokine signaling. This is particularly significant, as STAT1 and STAT3 are key transcription factors that mediate the expression of various pro-inflammatory genes, including IL-1β, IL-6, and iNOS [45,46]. Our findings are consistent with previous reports demonstrating that natural compounds can exert anti-inflammatory effects by decreasing STAT activation in macrophages [47]. Collectively, these results suggest that the anti-inflammatory effects of PCE are, at least in part, mediated through the suppression of phosphorylation of STAT in LPS-activated macrophages. These findings support the potential therapeutic application of PCE in the treatment of inflammation-related diseases.

### 2.7. PCE Inhibits Inflammation via AP-1 and MAPKs Pathway Suppression in Macrophages

**Figure 6:**
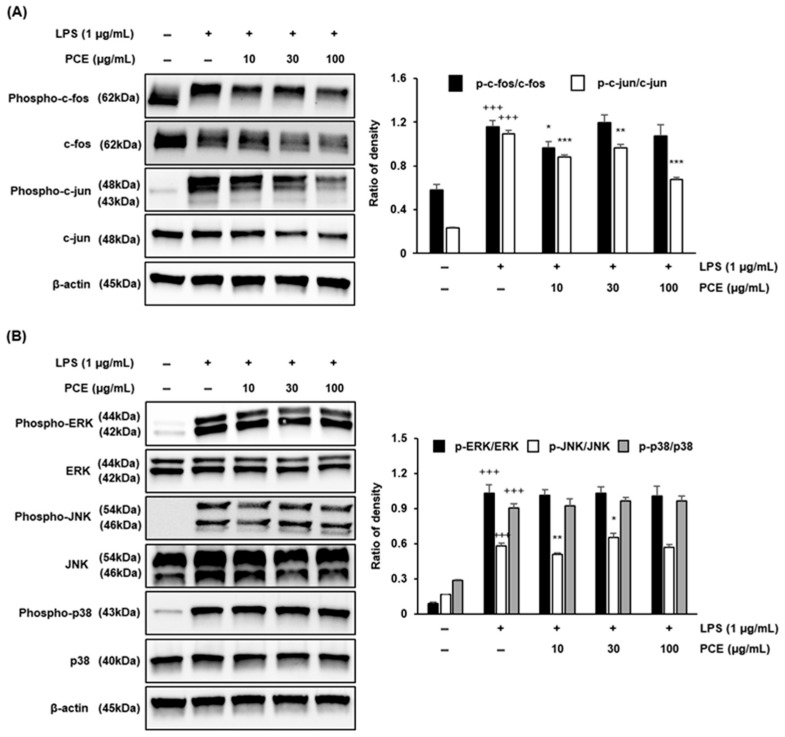
The effects of PCE on the expression of AP-1 (**A**) and MAPKs (**B**) were determined by Western blot analysis in LPS-stimulated RAW 264.7 macrophages. RAW 264.7 cells were cultured with LPS for 1 h before treatment with PCE (10, 30, and 100 μg/mL) for 3 h. For quantification, the expression data were normalized to the β-actin signal. Data are presented as mean ± SD from three independent experiments. Statistical significance was analyzed using one-way ANOVA followed by post hoc testing (* *p* < 0.05, ** *p* < 0.01, *** *p* < 0.001 vs. control; +++ *p* < 0.001 vs. LPS).

To elucidate the anti-inflammatory mechanisms of PCE, we investigated its effects on the activator protein-1 (AP-1) transcription factor and the mitogen-activated protein kinase (MAPK) signaling pathways in LPS-stimulated RAW 264.7 macrophages (Figure 6). As shown in Figure 6A, PCE treatment resulted in a dose-dependent decrease in the phosphorylation of c-Fos and c-Jun, key components of the AP-1 complex. Notably, at the highest concentration (100 μg/mL), PCE significantly suppressed phospho-c-Fos and phospho-c-Jun expression, indicating the inhibition of AP-1 activation. Since AP-1 regulates the transcription of pro-inflammatory genes such as COX-2, TNF-α, and iNOS [48,49], this finding supports the hypothesis that PCE modulates inflammation at the transcriptional level. To explore the molecular mechanisms further, we examined whether PCE affects upstream signaling pathways involved in AP-1 activation. As shown in Figure 6B, PCE treatment led to a significant reduction in JNK phosphorylation, a key regulator within the MAPK family. MAPK pathways, including JNK, are central to orchestrating inflammatory responses by regulating cytokine production and immune gene expression [13,50]. The inhibition of JNK phosphorylation by PCE suggests that its anti-inflammatory effects are mediated through interference with upstream signaling pathways regulating AP-1 activation. These findings highlight PCE’s potent anti-inflammatory activity through dual inhibition: the suppression of AP-1 activation and interference with the upstream JNK signaling cascade. This further supports the therapeutic potential of PCE as a natural compound for the prevention and treatment of inflammation-associated diseases.

## 3. Materials and Methods

### 3.1. Pretreatment and Extraction of Samples

The Young leaves of reed (*Phragmites communis*) samples were collected from Suncheon Bay, located at 160-13 Daedae-dong, Suncheon-si, Jeollanam-do, Republic of Korea (Latitude: 34°53′13.578″N; Longitude: 127°30′38.955″E), during June to July 2018.

The young leaves of *Phragmites communis* were thoroughly washed with purified water and air-dried at room temperature for 48 h. The extraction was performed by refluxing the dried leaves in ethanol (100 g/L) with continuous stirring at 600 rpm for 24 h at room temperature in a dark environment. The extract was sequentially filtered through standard filter paper, followed by a polypropylene membrane. The filtrate was concentrated under reduced pressure and subsequently freeze-dried to obtain the *Phragmites communis* extract (PCE), with a final yield of 1.48%. For in vitro experiments, the freeze-dried extract was dissolved in dimethyl sulfoxide (DMSO) to prepare a 300 mg/mL stock solution, which was stored at –20 ± 2 °C, ensuring that the final DMSO concentration in all cell-based assays did not exceed 0.1% (*v*/*v*).

### 3.2. Quantification of Condensed Tannins, Flavonoids, and Phenolics

Condensed tannin content was quantified using a modified vanillin assay, based on the reaction of vanillin with catechins and proanthocyanidins [51]. Tannin levels were expressed as catechin equivalents (CE) per kilogram of dry mass. A standard calibration curve was generated using catechin, and results were reported as μg CE/mg dry weight. Total flavonoid content was determined using an aluminum chloride colorimetric method with slight modifications from previously described protocols [52,53]. Quercetin was used as the standard compound to generate a calibration curve within the range of 1–500 μg/mL. Flavonoid content was expressed as μg quercetin equivalents (QE)/mg. Total phenolic content was assessed using a modified Folin–Denis method [54]. Gallic acid served as the standard for constructing the calibration curve over the concentration range of 1–500 μg/mL. Results are expressed as μg gallic acid equivalents (GAE)/mg of dry weight.

### 3.3. Cell Culture

RAW 264.7 murine macrophage cells were obtained from the Korean Cell Line Bank (Seoul, Republic of Korea). Cells were maintained in RPMI 1640 medium supplemented with 10% fetal bovine serum (FBS) and 1% penicillin–streptomycin (Gibco, ThermoScientific Co., Waltham, MA, USA). Cultures were incubated at 37 °C in a humidified atmosphere containing 5% CO_2_.

### 3.4. Cell Viability Assay

Cell viability was evaluated using the Cell Counting Kit-8 (CCK-8; Dojindo, Kumamoto, Japan) assay. RAW 264.7 cells were seeded in 96-well plates at a density of 2.5 × 10^5^ cells/well. After 24 h of incubation with 100 μL of test samples at 37 °C, 10 μL of CCK-8 solution was added to each well, followed by an additional 3 h incubation. Absorbance was measured at 450 nm using a microplate reader (VersaMax, Molecular Devices, Sunnyvale, CA, USA). Cell viability was calculated as a percentage of the untreated control using the following equation:

Inhibition (%) = [1 − (OD_sample/OD_control)] × 100

### 3.5. Measurement of Nitric Oxide (NO) Production

The accumulation of nitrite, a stable metabolite of nitric oxide (NO), was measured using the Griess reaction. RAW 264.7 macrophages were seeded in 96-well plates at a density of 5 × 10^4^ cells/well in 100 μL of complete medium and incubated overnight at 37 °C in a 5% CO_2_ atmosphere. Cells were pretreated with lipopolysaccharide (LPS, 1 μg/mL) for 1 h to induce inflammation, followed by treatment with various concentrations of test samples for 24 h.

After incubation, 100 μL of culture supernatant from each well was transferred to a new 96-well plate, and 100 μL of Griess reagent (0.1% N-1-naphthyl-ethylenediamine in H_2_O and 1% sulfanilamide in 5% phosphoric acid, mixed at a 1:1 ratio) was added. The mixture was incubated at room temperature for 10 min, and absorbance was measured at 550 nm using a microplate reader. Nitrite concentration was calculated using a sodium nitrite (NaNO_2_) standard curve and expressed in μM [55,56].

### 3.6. Measurement of Pro-Inflammatory Cytokines and Prostaglandin E_2_

RAW 264.7 macrophages were seeded in 24-well plates at a density of 5 × 10^5^ cells/well and incubated overnight at 37 °C in a 5% CO_2_ humidified incubator. Cells were then treated with lipopolysaccharide (LPS, 1 μg/mL) and various concentrations of test samples for 24 h. After incubation, culture supernatants were collected to quantify cytokines and prostaglandin E_2_ (PGE_2_).

Levels of IL-6, TNF-α, and GM-CSF in the supernatants were determined using commercial enzyme-linked immunosorbent assay (ELISA) kits according to the manufacturer’s protocols. The detection limits for each cytokine were 3.8 pg/mL for IL-6, 1.07 pg/mL for TNF-α, and 15.6 pg/mL for GM-CSF, as reported by the kit manufacturers, ensuring the sensitivity and reliability of the measurements. Briefly, 96-well plates were coated with capture antibodies diluted in coating buffer (100 μL/well) and incubated overnight at 4 °C. Plates were washed and blocked with phosphate-buffered saline (PBS) containing 10% FBS. Diluted culture supernatants were added to each well and incubated at room temperature. After washing, biotin-conjugated detection antibodies (100 μL/well) were added and incubated, followed by HRP-conjugated enzyme reagent. Substrate solution was added to develop color, and absorbance was measured using a microplate reader.

PGE_2_ levels were measured using a Prostaglandin E_2_ parameter assay kit (R&D Systems, Minneapolis, MN, USA), with a reported detection limit of 39.0 pg/mL, in accordance with the manufacturer’s instructions. Cytokine concentrations were calculated from standard curves and expressed in pg/mL. PGE_2_ levels were calculated using a four-parameter logistic (4-PL) regression model [55,56].

### 3.7. Western Blot Analysis

Cultured RAW 264.7 cells were harvested and washed three times with PBS, followed by lysis using radio-immunoprecipitation assay (RIPA) buffer. Cell lysates were centrifuged at 15,000 rpm for 30 min at 4 °C, and the supernatant was collected. The total protein concentration was determined using a BCA protein assay kit (Pierce Biotechnology, Rockford, IL, USA). Equal amounts of protein (20 μL) were mixed with 4× Laemmli sample buffer, denatured at 100 °C for 5 min, and separated by 10% SDS-PAGE.

Following electrophoresis, proteins were transferred onto polyvinylidene fluoride (PVDF) membranes and blocked with 3% non-fat dry milk in TBS-T for 1 h at room temperature. Membranes were then incubated overnight at 4 °C with primary antibodies against iNOS (sc-7271), COX-2 (sc-376861), p38 (sc-7972), phospho-p38 (sc-7973), ERK (sc-1647), phospho-ERK (sc-7383), JNK (sc-7345), phospho-JNK (sc-6254), IκBα (sc-1643), phospho-IκBα (sc-8404), IKKα/β (sc-34673), phospho-IKKα/β (sc-21661), STAT1 (sc-398524), phospho-STAT1 (sc-8394), STAT3 (sc-293151), phospho-STAT3 (sc-8059), and β-actin (sc-47778), all purchased from Santa Cruz Biotechnology (Santa Cruz, CA, USA) and used at a dilution of 1:1000. After washing, HRP-conjugated goat anti-rabbit secondary antibodies were applied.

Protein bands were visualized using enhanced chemiluminescence (ECL) reagents and detected with the ChemiDoc imaging system (Bio-Rad, Hercules, CA, USA). Band intensities were quantified using Image Lab software 6.0 (Bio-Rad) and normalized to β-actin. The relative expression levels were calculated with reference to the LPS-treated control group [57].

### 3.8. Statistical Analysis

For the purpose of statistical analysis, all experiments were repeated in at least three independent trials. All experiments were performed in triplicate, and data are presented as means ± standard deviation (S.D.). Statistical analyses were conducted using one-way analysis of variance (ANOVA) with the Statistical Analysis System (SAS) version 9.4 (SAS Institute Inc., Cary, NC, USA). Differences between means were determined using the Duncan multiple-range test, with significance set at *p* < 0.05.

## 4. Conclusions

In this study, we identified ethanol extracts of PCE as a promising natural compound with potent anti-inflammatory activity in LPS-stimulated RAW 264.7 macrophages. The extract significantly inhibited the expression of key pro-inflammatory mediators, including inducible nitric oxide synthase (iNOS) and cytokines such as TNF-α, IL-6, and GM-CSF, as demonstrated by ELISA and Western blot analyses.

Mechanistically, PCE modulated multiple signaling pathways involved in inflammation. Specifically, it suppressed the phosphorylation and degradation of IκBα, thereby inhibiting the activation of NF-κB. Additionally, PCE reduced the phosphorylation of JNK and ERK, which are key regulators of the AP-1 transcription factor. The extract also interfered with the STAT1/3 signaling cascade, further supporting its broad-spectrum inhibitory effect on inflammation-related signaling.

These findings provide preliminary insight into the potential of PCE as a multi-targeted anti-inflammatory agent. The observed ability to modulate several key inflammatory pathways suggests that PCE merits further investigation for its potential application in treating chronic inflammatory diseases, where pathway redundancy often limits the effectiveness of single-target therapies. However, the absence of standard comparators in this study is acknowledged as a limitation, making it difficult to directly assess the relative efficacy and relevance of the observed effects. Moreover, given the exclusive reliance on in vitro data, these results should be interpreted with caution. Additional in vivo and clinical studies are necessary to validate these findings, confirm safety profiles, and better assess the translational relevance of PCE. The absence of apoptosis and necrosis assays is also recognized as a limitation, and these analyses are planned for future studies to further substantiate the observed effects. Furthermore, future research will incorporate comprehensive phytochemical profiling—including preliminary techniques such as TLC, UV spectroscopy, and general class tests—to enhance the interpretability, significance, and reliability of the results. Future investigations may also consider re-evaluating tannin profiles using alternative extraction methods to potentially uncover additional bioactive components. Nonetheless, our study highlights the promise of plant-derived compounds like PCE as viable candidates for future inflammation research.

## Figures and Tables

**Table 1:** The chemical quantification of *Phragmites communis* leaf extract (PCE).

Type of Sample	Polyphenols (GAE) ^a^	Flavonoids (QE) ^b^
PCE	4.14 ± 0.60	10.18 ± 1.29

Data are the mean ± S.D. of three independent measurements. ^a^ microgram gallic acid equivalents per milligram; ^b^ microgram quercetin equivalents per milligram.

## Data Availability

Data are contained within the article.
